# *Portulaca oleracea* L. aids calcipotriol in reversing keratinocyte differentiation and skin barrier dysfunction in psoriasis through inhibition of the nuclear factor κB signaling pathway

**DOI:** 10.3892/etm.2014.2116

**Published:** 2014-12-08

**Authors:** HENGGUANG ZHAO, SHUANG LI, FULING LUO, QIAN TAN, HUI LI, WEIKANG ZHOU

**Affiliations:** 1 Department of Dermatology, The First Affiliated Hospital of Chongqing Medical University, Chongqing 400016, P.R. China; 2 Department of Dermatology, Chongqing Third People’s Hospital, Chongqing 400014, P.R. China; 3 Department of Pharmacy, The First Affiliated Hospital of Chongqing Medical University, Chongqing 400016, P.R. China

**Keywords:** calcipotriol, nuclear factor κB pathway, *Portulaca oleracea* L., psoriasis, skin barrier function

## Abstract

Psoriasis affects 2–4% of the population worldwide and its treatment is currently far from satisfactory. Calcipotriol and *Portulaca oleracea* have been reported to exhibit the capacity to inhibit inflammation in psoriatic patients and improve their clinical condition. However, the efficacy of a combination regimen of these two components remains unknown. The aim of the present study was to explore the therapeutic efficacy of *P. oleracea* extract combined with calcipotriol on plaque psoriasis and its potential mechanism. Eleven patients with plaque psoriasis were treated with humectant containing the active ingredients of *P. oleracea* extract, with or without 0.005% calcipotriol ointment in a right-left bilateral lesion self-control study. Differences were evaluated by investigation of the clinical efficacy, adverse effects, skin barrier function, histological structure, expression and proliferation of keratinocytes, differentiation markers (cytokeratin 10, filaggrin and loricrin), inflammatory factors [tumor necrosis factor (TNF)-α and interleukin (IL)-8], as well as the status of the nuclear factor κB (NF-κB) pathway. The combination of *P. oleracea* and calcipotriol was revealed to decrease adverse effects, reduce transepidermal water loss, potently reverse keratinocyte differentiation dysfunction, and inhibit the expression of TNF-α and IL-8 and the phosphorylation of the NF-κB inhibitor IκBα. This treatment is therefore anticipated to be suitable for use as a novel adjuvant therapy for psoriatic patients.

## Introduction

Psoriasis is a chronic inflammatory skin disease characterized by epidermal hyperproliferation and altered differentiation, with a prevalence of 2–4% worldwide (1). Chronic plaque psoriasis, or psoriasis vulgaris, is the most common form of the disease with well a circumscribed erythematous and indurated plaque scale, accounting for 85–90% of cases. At present, there is no curative therapy available to fully treat the disease, and the typical clinical course is of chronic relapse and remission (2). Previous studies support a pivotal role for nuclear factor κB (NF-κB) activation in the pathogenesis of psoriasis (3). The overactivation of NF-κB in the psoriatic epidermis has been hypothesized to induce altered keratinocyte proliferation and differentiation (4). Increased activation may cause NF-κB to translocate into the nucleus and subsequently promote the transcription of target gene sequences, including the keratinocyte differentiation markers of cytokeratin 10 (K10), cytokeratin 16 (K16), loricrin (LOR) and filaggrin (FLG) (5–8), and also regulate the cell cycle, which is considered to be significantly accelerated in the pathogenesis of psoriasis (9). Various reagents that are capable of regulating the status of the NF-κB pathway have been used to treat psoriasis. Calcipotriol is notable among these. A study has demonstrated that calcipotriol may regulate the NF-κB pathway through inversing the binding activation of NF-κB to its target gene response elements, including p53 and interleukin (IL)-8 (10) and subsequently regulating their transcription and protein expression. Calcipotriol has shown clear therapeutic effects on psoriasis vulgaris and is widely used in the majority of countries. However, clinical studies have reported that calcipotriol may simultaneously induce clear adverse effects, including impairment of the skin barrier function and evident irritation to the psoriatic skin, particularly following long-term topical application (11–13).

*Portulaca oleracea* L. (purslane) is a green plant and vegetable consumed mainly in the eastern Mediterranean region, and is also commonly known as machixian in China and pursley in the USA. In ancient China, it was medically used as an effective cure for blasting and burning by gunpowder, and it has also been used as a folk medicine in a number of other countries to treat various ailments in humans, including as a cooling diuretic, refrigerant and tonic, as well as an article of diet used to treat scurvy, liver complaints, sore nipples, stomach and mouth ulcers, and for reducing inflammation (14). Modern studies have revealed that *P. oleracea* leaves are a rich source of linolenic acid (LNA) and α-tocopherol (α-TCP) (15,16), and its extracts are capable of regulating the tumor necrosis factor-α (TNF-α)-induced NF-κB signaling pathway (17) as well as suppressing the overexpression of proinflammatory factors, including vascular cell adhesion molecule-1, intercellular adhesion molecule-1, E-selectin, matrix metalloproteinase-2 (17) and transforming growth factor-β1 (18). In dermatology, the fresh crude extract of *P. oleracea* has been reported to significantly stimulate physical wound contraction and accelerate the wound healing process by decreasing the surface area and increasing the tensile strength of the skin (19,20), as well as by inhibiting mushroom tyrosinase, indicating that it may be used to inhibit tyrosinase in skin, resulting in repression of the synthesis of melanin pigments and playing a crucial protective role against skin photocarcinogenesis (21).

In the present study, a clinical right-left bilateral lesion self-control study was performed to explore the efficacy of *P. oleracea* with or without calcipotriol in psoriatic patients, This involved a comparison between the crude extracts of *P. oleracea* (Winona; Kunming Dihon Pharmaceutical Co., Ltd., Kunming, China) in combination with 0.005% calcipotriol ointment (Daivonex^®^; LEO Laboratories Ltd., Dublin, Republic of Ireland) and monotherapy with 0.005% calcipotriol ointment alone, which was approved in accordance with the ethical committee approval process of the First Affiliated Hospital of Chongqing Medical University (Chongqing, China).

## Materials and methods

### Human subjects

The present study was conducted as a single-center, prospective, bilateral comparison study. Written informed consent was obtained from each patient prior to enrolment in the study. A total of 11 Chinese patients with plaque psoriasis (seven males and four females), aged 21.3–47.1 years (mean age 32.4 years), were recruited. For each individual, it was required that at least two target lesion pairs on each side of the body were of moderate to severe severity, and all comparative lesion pairs had to be in analogous anatomic locations and of approximately equal severity. Scalp, facial and genital psoriasis was neither treated nor assessed. Those patients who had received oral, topical, physical (i.e., ultraviolet or solarium treatment) and systemic antipsoriatic treatments within the previous six months were excluded. Other exclusion criteria included a current diagnosis of unstable psoriasis, pregnancy, benign or malignant tumor, topically serious skin trauma, uncontrollable systemic disease and a history of allergy.

Calcipotriol ointment alone was applied twice daily to the affected areas on one side of the body [monotherapy (M)group], randomly assigned for each patient. On the other side, calcipotriol ointment was applied only once in the evening and humectant containing *P. oleracea* extract once in the morning rotationally [combination (C) group]. Due to ethics restrictions, *P. oleracea* treatment alone could not be applied during the study. A fixed dosage was strictly required at a total of 0.5 g (a fingertip unit (22)) on each 10×10 cm^2^ area for each application, which was calculated and strictly instructed by the investigators at the first visit of the patient. The therapeutic phase of the sides was equal at four weeks.

### Clinical assessments

The severities of the paired psoriatic lesions were recorded on each visit (weeks 0, 2 and 4).Scales, plaque and erythema were usually considered as the main complaints of psoriasis. A nine-value rating scale with 0.5-point increments was used to evaluate the change in the degree of scales, plaque elevation and erythema, with the following classifications: 0, none; 1, mild; 2, moderate; 3, severe; and 4, very severe (23). Total scores were calculated as the sum of the points. Another assessment of overall efficacy was made by the patients using the following defined five-point grading scale on each visit: 1, excellent improvement (>75%); 2, marked improvement (51–75%); 3, moderate improvement (26–50%); 4, slight improvement (0–25%); and 5, no improvement or deterioration. Symptoms including itching, thermalgia, as well as any another abnormal sensations or adverse events, if any, were documented in detail on each visit.

### Transepidermal water loss (TEWL) measurement

To evaluate the condition of the skin barrier, patients were investigated instrumentally for TEWL, using a Tewameter^®^ TM 300 (Courage + Khazaka electronic GmbH, Cologne, Germany) under the manufacturer’s instructions. The condition of all subjects was first stabilized for 15–20 min, in a climate- and humidity-controlled room, with an ambient temperature range of 21–25°C and mean relative humidity range of 50–60%. Two topical comparative plaques of homologous anatomic positions and severity on each side were measured at each visit at weeks 0, 2 and 4. Additionally, five healthy volunteers were recruited as the control.

### Tissue collection

Skin tissues for downstream analysis were obtained from each patient at weeks 0 and 4 by a professional operator. Samples were approximately equally harvested on the bilateral body each time, and the regions at week 4 were close to those of week 0 while avoiding the scar. The tissues were fixed immediately according to the different downstream processes. Additionally, another five skin tissues without substantial lesions were obtained from the Department of Surgery in The First Affiliated Hospital of Chongqing Medical University as the normal control.

### Hematoxylin and eosin staining (H&E) and epidermal thickness measurement

Routine H&E staining was conducted following paraffin sectioning of 5-μm-thick sections. The mean epidermal thickness was determined on H&E stained sections by measuring the distance between the outermost surface of the epidermis excluding the stratum corneum and the dermo-epidermal junction at five points through the entire length of three examined sections for each specimen.

### Immunohistochemistry (IHC)

To perform IHC, the slides were retrieved in a high-temperature antigen retrieval solution of citrate buffer and then incubated overnight at 4°C with primary antibodies (Abs) against K10, LOR, FLG (Abcam, Cambridge, MA, USA), TNF-α and IL-8 (Cell Signaling Technology, Inc., Danvers, MA, USA). Subsequently, the appropriate secondary Abs and detection kits were used. The tissues were visualized with 3,3′-diaminobenzidine substrate and counterstained with hematoxylin. Images were captured under closely comparable conditions.

### Western blot analysis

Skin tissues from patients were homogenized and sonicated in lysis buffer (containing 20 mM Tris, 150 mM NaCl, 1 mM EDTA, 1 mM ethylene glycol tetraacetic acid, 1% Triton, 0.1% sodium dodecyl sulfate (SDS) and 1% protease inhibitors). Equal amounts of proteins were separated on 7–12% SDS-polyacrylamide gels in a minigel apparatus (Bio-Rad, Hercules, CA, USA) and then transferred electrophoretically to nitrocellulose membranes, followed by blocking with milk and incubation at 4°C overnight with anti-K10, LOR, FLG, TNF-α and IL-8 Abs, as well as anti-p65, phosphorylated-(p-)p65, inhibitor κBα (IκBα) and p-IκBα (phosphorylated at Ser32) Abs (Cell Signaling Technology, Inc.). Membranes were incubated for 1 h with horseradish peroxidase-conjugated secondary Abs. Subsequent to immunoblotting, the films were scanned and the intensity of the immunoblotting bands was detected with a Bio-Rad GS-800 Calibrated Densitometer (Bio-Rad). GAPDH and α-tubulin were used as the loading controls.

### Statistical analysis

Severity scores from the clinical assessments were compared at the baseline and across three time points using the Wilcoxon signed-rank test. The comparisons of the various severity scores between the M group and C group-treated lesion pairs were evaluated using the Mann-Whitney U test, and comparison of the adverse effects between the two groups was conducted using the Pearson’s χ^2^ test. The results of the readings of TEWL, epidermis thickness and quantitative western blot images are all presented as the mean ± standard deviation, and were analyzed using the two-tailed unpaired Student’s t-test or paired-samples Student’s t-test. All analyses were performed using SPSS version 13.0 for Windows (SPSS, Inc., Chicago, IL, USA). P<0.05 was considered to indicate a statistically significant difference.

## Results

### P. oleracea in combination with calcipotriol effectively improves the clinical manifestations to the same extent as calcipotriol monotherapy, but with fewer adverse effects

Ten patients (78 target lesion pairs) that completed the phase of treatment were included in the statistical analysis for efficacy. The locations of the target lesions were on the legs (n=37), arms (n=24) and trunk (n=17). One patient dropped out due to rejection to the second skin biopsy at week 4, when the clinical manifestation had improved markedly.

**Figure 1:**
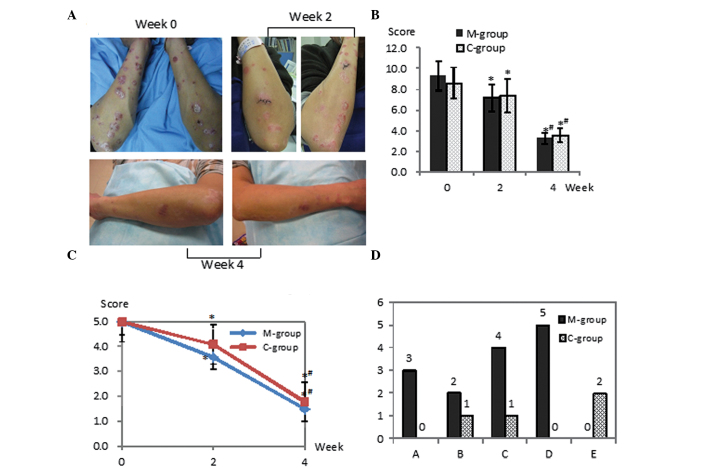
Clinical improvement prior and subsequent to treatments with the two regimens. (A) Representative images from one patient in this study, whose left arm was treated with the calcipotriol ointment alone and the right with humectant containing *Portulaca oleracea* extract in combination with calcipotriol. (B) The statistical analysis of the scores representing the severity of the scales, plaque elevation and erythema. (C) The statistical analysis of the overall efficacy assessment evaluated by the patients. (D) Comparison of the adverse effects between the two groups (P<0.05): A, temporary skin redness; B, itching; C, transient burning sensation; D, skin dryness; E, aromatic odor. ^*^P<0.05 compared with week 0; ^#^P<0.05 compared with week 2. M group, monotherapy group; C group, combination group.

At the baseline, the severity of lesions between the two groups was similar in scaling, plaque elevation, erythema and overall lesional assessment. Subsequent to treatment, the two groups showed statistically significant amelioration in scaling, plaque elevation and erythema, as well as in the overall lesional severity, which was estimated by the patients themselves at weeks 2 and 4 (Fig. 1A–C). Comparing the two regimens, no statistically significant difference was identified at the two visits. However, according to the reports of the patients, the adverse effects in the M group were of significantly higher frequency than those in the C group during the first two weeks (P<0.05), including five individual complaints of skin dryness and four of transient burning sensation in the applied area, as well as three and two complaints of immediate temporary skin redness and/or itching following the application in the M group. However, all these complaints decreased gradually and were eventually eliminated in two weeks. By contrast, in the C group, the main complaints were the slightly aromatic odor of the humectant, an occasional slight burning sensation and itching (Fig. 1D).

### Combination regimen improves skin barrier function with greater efficiency than calcipotriol monotherapy

At the beginning of the study, the TEWL of the patients was significantly higher than that of the normal controls, which indicated the impaired function of the skin barrier in the psoriatic lesions. In the M group, the TEWL was slightly lower following treatment than the initial TEWL at weeks 2 and 4; however, no statistically significant differences were identified, indicating that the calcipotriol ointment was not able to protect against water loss and improve the skin barrier function in the present study. By contrast, the value of TEWL in the C group was significantly decreased at the visits at weeks 2 and 4, accompanied by the normalization of the lesional condition. When compared with the M group, this clearly indicated that the improvement of skin barrier function with the combination regimen was more efficient than that with the monotherapy of calcipotriol (Table I).

### Combination therapy has a higher efficacy than calcipotriol monotherapy for attenuating the abnormal differentiation of psoriatic keratinocytes

**Figure 2:**
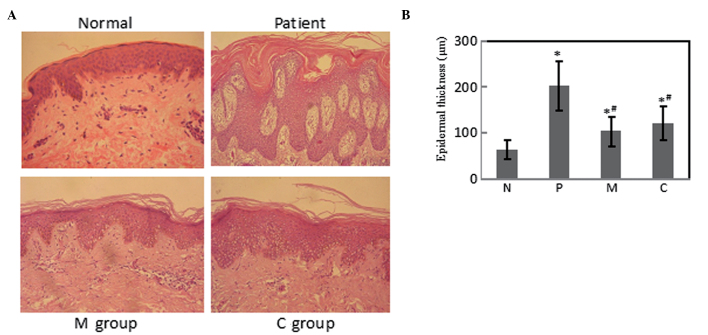
Hematoxylin and eosin staining of psoriatic lesions and comparative measurement of epidermal thickness. (A) Epidermal hyperplasia with thickened keratin layers was clearly indicated in the psoriatic epidermis prior to treatment, with the rete ridges narrowly lifted towards the surface and broadened at the base. Following treatment, with the improvement of the clinical presentations, all the histological landmarks were ameliorated synchronously. (B) The epidermal thickness was markedly increased in the patients pre-treatment and decreased at week 4 of the treatments. ^*^P<0.05 compared with the normal control; ^#^P<0.05 compared with prior to treatment. M, monotherapy; C, combination; N, normal control; P, psoriatic samples prior to treatment.

To evaluate the keratinocyte proliferation status, epidermal thickness was investigated by routine H&E staining (Fig. 2). The results clearly showed that prior to treatment there was a pronounced increase in the epidermal thickness with the acanthotic appearance and rete ridges, accompanied by abnormal keratinocyte differentiation with the loss of the stratum granulosum. The stratum corneum was clearly thickened with parakeratosis. Subsequent to treatment, however, the layers of keratinocytes decreased along with the flattened rete ridges and the stratum granulosum cell layers increased. Comparison between the two regimens indicated that although the change of epidermal thickness was slightly greater in the M group than in the C group, no significant difference was statistically validated.

**Figure 3:**
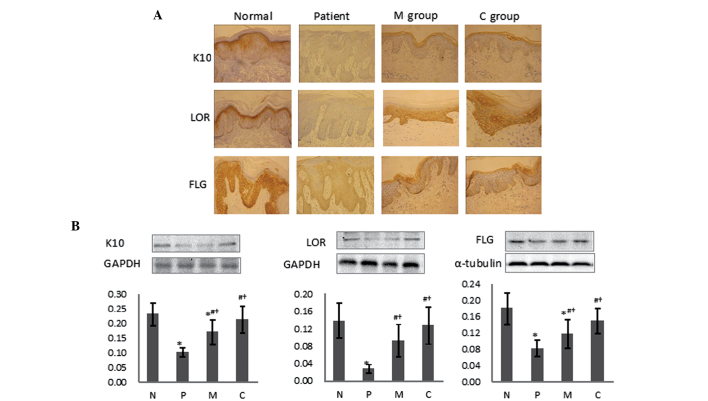
Expression of the markers relevant to keratinocyte differentiation verified by immunohistochemistry and western blot analysis. (A) Images of immunohistochemical staining (magnification, ×200). The images are representative of those obtained from the subjects. (B) Expression levels of the proteins associated with keratinocyte differentiation were evaluated by western blotting. Images are representative of those obtained from the subjects and were quantitatively analyzed using the appropriate software. M and C samples were obtained at week 4 following treatment. ^*^P<0.05 compared with the normal control; ^#^P<0.05 compared with prior to treatment; ^†^P<0.05 compared with the other treatment group following treatment. GAPDH and α-tubulin served as loading controls. N, normal control; P, psoriatic samples prior to treatment; M, monotherapy; C, combination.

To further explore the keratinocyte differentiation status, the expression of protein markers reflecting the differentiation of the psoriatic keratinocytes was then investigated by IHC (Fig. 3A) and western blot analysis (Fig. 3B). These revealed that the K10 protein was mainly expressed in the stratum spinosum at moderate levels in normal tissue, while no expression was found in skin psoriatic lesions; however, K10 protein expression was strongly elevated following treatment in the two treatment groups. A notable quantity of LOR was detected in the stratum granulosum and lower corneum, which was significantly downregulated in the psoriasis lesions; however, evident elevation was detected following treatment. FLG expression of a moderate level was detected in normal controls and a lower expression was observed in untreated patients, with upregulation following treatment. Paralleled with the immunohistological outcomes, western blotting also revealed the same tendencies for the normal tissue, psoriatic skin and those following treatment. The protein expression levels of K10, LOR and FLG were significantly downregulated in the psoriatic lesions. However, following treatment, all these changes in protein expression levels were significantly reversed and accompanied by an improvement in skin condition. The combination therapy exerted markedly higher efficacies on inversing the abnormal expression levels of these differentiation markers for psoriatic keratinocytes.

### TNF-α and IL-8 expression is downregulated significantly more evidently by combination therapy than by monotherapy

**Figure 4:**
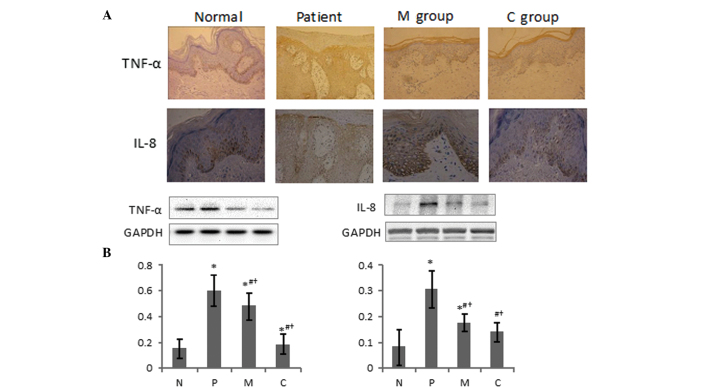
Differential expression levels of the inflammatory factors TNF-α and IL-8 investigated by immunohistochemistry and western blot analysis. (A) Images of immunohistochemical staining (magnification, ×200). Images are representative of those obtained from the subjects. (B) Expression levels of TNF-α and IL-8 were evaluated by western blotting. Images are representative of those obtained from the subjects and were quantitatively analyzed using the appropriate software. TNF-α and IL-8 were significantly upregulated in psoriatic tissue compared with normal tissue, and markedly decreased following the treatments. The reversal efficiency in the C group was higher than that of the M group. M and C samples were obtained at week 4 following treatment. ^*^P<0.05 compared with the normal control; ^#^P<0.05 compared with prior to treatment; ^†^P<0.05 compared with the other treatment group following treatment. GAPDH served as a loading control. N, normal control; P, psoriatic samples prior to treatment; M, monotherapy; C, combination.

TNF-α and IL-8, two pivotal inflammatory factors according to previous reports (3,24,25), are both involved in the pathogenesis of psoriasis, and have been proposed to be the crucial therapeutic targets for psoriatic patients. To explore if these factors were differentially regulated by the two regimens, their expression levels were investigated by IHC and western blot analysis. Fig. 4A shows that TNF-α and IL-8 expression was at a limited level and mainly distributed to the basal layers of the epidermis in normal tissue, but was widely upregulated within the whole epidermis in the psoriasis tissues, while being significantly decreased following treatment with the two regimens. Western blot analysis revealed a comparable tendency, as shown in Fig. 4B. However, following treatment, a significant difference between the effects of the combination therapy and monotherapy was statistically concluded for the TNF-α and IL-8 expression levels by quantitative analysis, thus revealing that the combination regimen exerted a more potent regulatory effect on these two inflammatory factors.

### P. oleracea and calcipotriol reverse keratinocyte dysfunction in psoriasis by repression of the NF-κB signaling pathway via a different pathway than calcipotriol alone

**Figure 5:**
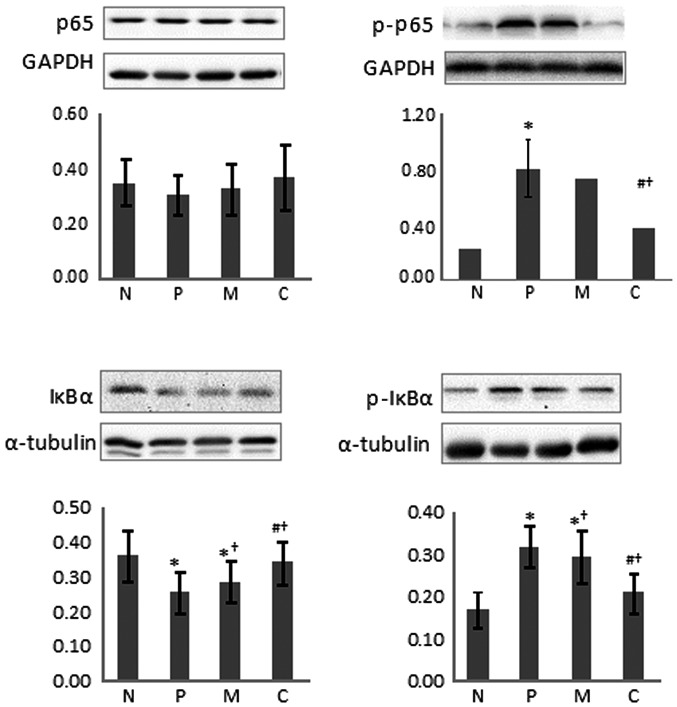
*Portulaca oleracea* aided calcipotriol in improving the psoriatic condition through inhibition of the nuclear factor-κB pathway (western blot analysis). Images are representative of those obtained from the subjects and were quantitatively analyzed using the appropriate software. No significant difference in p65 was identified between the two regimens. IκBα was notably decreased and p-p65 and p-IκBα were increased in the psoriatic lesions prior to treatment, which was significantly reversed following the treatment of the C group, but not the calcipotriol M group at week 4 following treatment. ^*^P<0.05 compared with normal control; ^#^P<0.05 compared with prior to treatment; ^†^P<0.05 compared with the other treatment group following treatment. GAPDH and α-tubulin served as the loading controls. N, normal control; P, psoriatic samples prior to treatment; M, monotherapy; C, combination; IκBα, inhibitor κBα; p-, phosphorylated-.

To further explore the potential mechanism by which *P. oleracea* aided calcipotriol in reversing the psoriatic condition, the status of the NF-κB pathway was investigated by western blot analysis. Since p65 is the main functional subunit of NF-κB, and its activation is mainly regulated by its inhibitory protein, IκBα (26,27), the expression levels of p65, p-p65, IκBα and p-IκBα were investigated. Fig. 5 shows that the expression levels of p65 were not significantly different between either the psoriatic lesions and normal controls, or prior and subsequent to treatment. However, the expression levels of p-p65 and p-IκBα were markedly increased, and IκBα levels were decreased in the psoriatic patients compared with those in normal tissue. Following treatment, the p-p65 and p-IκBα levels were decreased significantly, and subsequently the level of IκBα was reversed in the C group, but not in the calcipotriol M group. For the calcipotriol M group, no changes in the expression of p-p65, IκBα and p-IκBα were identified following treatment, which was consistent with a previous study (9), and therefore it was proposed that the combination regimen but not the calcipotriol directly suppressed the degradation of IκBα in the present study, which subsequently resulted in the accumulation of IκBα and inhibited the NF-κB activation and nuclear translocation.

## Discussion

Calcipotriol, a vitamin D analog, is one of the most common therapies for topical psoriasis lesions. It has been reported to effectively attenuate the abnormal differentiation and proliferation of psoriatic keratinocytes (28–31), but simultaneously arouse clear adverse effects, including impairment of the skin barrier function and the induction of irritating erythema and itching, particularly following long-term topical application (11–13). Therefore it is of great significance to explore novel potential adjuvants or combinative regimens that are capable of not only enhancing the efficacy of treatment but also reducing the adverse reactions. *P. oleracea* L., a traditional Chinese herb once used to treat certain dermatological diseases in traditional Chinese medicine, was used in combination with calcipotriol in the present study to treat topical psoriasis. The findings showed that extracts of *P. oleracea* clearly aided calcipotriol in improving the condition of psoriasis lesions, together with markedly alleviating adverse effects compared with calcipotriol monotherapy, and thus revealed a potential therapeutic efficiency in psoriatic patients by the lower dosage of calcipotriol required and its slighter irritation to the skin.

In the combination regimen, the skin barrier dysfunction was effectively reversed. TEWL, one of the most important parameters reflecting the skin barrier function, was verified to be clearly impaired in the psoriatic lesions prior to treatment and significantly improved in the C group but not the calcipotriol M group, demonstrating that the combination regimen with *P. oleracea* could further aid the reversal of the skin barrier dysfunction compared with the calcipotriol monotherapy. The skin barrier is mainly dependent on the integrity and normal differentiation of keratinocytes, the intercellular matrix and superficial cuticles on the epidermis (32). To evaluate the condition of psoriatic keratinocyte proliferation and differentiation, the epidermal thickness and the differential markers K10, LOR and FLG were investigated. It was shown that the combination and monotherapy regimens evidently repressed the proliferation of psoriatic keratinocytes. Although the reduction of epidermal thickness was slightly higher in the monotherapy than the combination regimen, no statistical significant difference was revealed. The expression levels of K10, LOR and FLG indicated by immunohistochemistry, paralleled with the western blot analysis, revealed that all the differentiation markers were significantly downregulated in psoriatic compared with normal tissue, which revealed the impaired differentiation properties of keratinocytes were involved in the pathophysiology of psoriasis. Furthermore, following treatment, the reversion of K10, LOR and FLG was more effective in the combination regimen than in the monotherapy, which clearly demonstrated that *P. oleracea* was capable of aiding calcipotriol in attenuating the severity and extent of psoriatic lesions, and that the underlying mechanism may partially be through regulation of the differentiation dysfunction of keratinocytes.

Furthermore, to explore the potential mechanism by which *P. oleracea* regulated the psoriatic keratinocyte differentiation, the levels of the inflammatory factors TNF-α and IL-8, which have been reported to play crucial roles in psoriatic pathogenesis, were investigated (24,25). The results showed that TNF-α and IL-8 were significantly upregulated in psoriatic compared with normal tissue, and markedly decreased following the treatments. However, the reversion efficiency for the combination therapy was higher than that for the calcipotriol monotherapy. This not only indicated that these inflammatory factors were involved in the psoriatic pathophysiology, but also that *P. oleracea* exhibits properties that aid calcipotriol in regressing the inflammation in psoriasis lesions, which is consistent with a previous study of the effect of *P. oleracea* on human disease (33).

Ongoing studies have disclosed that calcipotriol regulates the target gene transcription through the NF-κB signal pathway by adjusting the binding activation of NF-κB to its target gene promoters, including TNF, IL-8 and IL-1α (24,25), but without the property of affecting the phosphorylation of IκB (9). The conditions of the pivotal proteins within the NF-κB pathway were investigated by western blot analysis. No expression level changes were revealed for p65 between the psoriatic lesions and normal controls. However, the expression level of IκBα was decreased and those of p-IκBα and p-p65 increased markedly in the psoriatic lesions, disclosing that activation of the NF-κB pathway was involved in the pathogenesis of psoriasis and that the hyperfunction of the NF-κB signal was induced, at least partially, by the decreased phosphorylation of IκB but not the increased levels of p65. Furthermore, following treatment, the expression level of p65 was not significantly changed by the treatment regimens, but the expression levels of p-p65 and p-IκBα were significantly decreased, and subsequently the level of IκBα increased in the C group but not in the calcipotriol M group, indicating that the NF-κB pathway was not repressed by calcipotriol but by *P*. *oleracea* (however, due to the ethics restrictions, *P. oleracea* treatment alone could not be administered). This finding is in accordance with an earlier study in which Johansen *et al* (10) showed that the application of calcipotriol was not capable of regulating the phosphorylation of IκBα and IκBβ, but normalized the abnormal NF-κB binding activity to certain gene promoter sites including p53 and IL-8. This lead to the hypothesis that *P. oleracea* may aid calcipotriol in repressing inflammatory factors and reversing the impaired differentiation of psoriatic keratinocytes through different pathways within the NF-κB signaling pathway, but with a reciprocal synergy.

In conclusion, the combination of calcipotriol and *P. oleracea* extracts may not only decrease the required dosage of calcipotriol and reduce its adverse effects, but also protect the skin barrier function and benefit the remedy of the impaired proliferation and differentiation of the psoriatic keratinocytes. It is speculated that the potential mechanism may lie in the mutual complementarity of the pharmacological effects of the two components within the same NF-κB pathway, in which calcipotriol inhibited NF-κB binding to the relevant gene promoter while *P. oleracea* decreased the phosphorylation of IκB and subsequently inhibited its degradation. As a rich source of LNA and α-TCP, *P. oleracea* is capable of further remedying the impaired intercellular matrix and decreasing the water loss and TEWL. Therefore, it is feasible and advisable that *P. oleracea* extracts may be administered as an efficient adjuvant reagent with calcipotriol application for psoriasis, which may lead to a novel auxiliary treatment for this chronic inflammatory skin disease.

## Figures and Tables

**Table I:** Comparison of transepidermal water loss between the two treatment groups and healthy controls.

Group	Week 0	Week 2	Week 4
Healthy controls (n=5)	9.46±2.27		
M group (n=10)	22.17±4.51^a^	20.93±4.05^a^,^b^	18.78±3.94^a^,^b^
C group (n=10)	21.63±4.85^a^	17.17±3.82^a^–^c^	13.46±3.73^a^–^c^

a P<0.05 compared with the normal controls,

b P<0.05 compared with the other treatment group and

c P<0.05 compared with prior to treatment.

Data are presented as the mean ± standard deviation (g/hm^2^). M group, monotherapy group; C group, combination group.
